# Since 2015 the SinoGerman research project SIGN supports water quality improvement in the Taihu region, China

**DOI:** 10.1186/s12302-016-0092-7

**Published:** 2016-10-27

**Authors:** Kathrin Rachel Schmidt, Tim aus der Beek, Xiaohu Dai, Bingzhi Dong, Elke Dopp, Florian Eichinger, Monika Hammers-Wirtz, Regina Haußmann, Andreas Holbach, Henner Hollert, Marc Illgen, Xia Jiang, Jan Koehler, Stephan Koester, Andreas Korth, Stephan Kueppers, Aili Li, Matthias Lohmann, Christian Moldaenke, Stefan Norra, Boqiang Qin, Yanwen Qin, Moritz Reese, Edmund Riehle, Beatrix Santiago-Schuebel, Charlotte Schaefer, Anne Simon, Yonghui Song, Christian Staaks, Joerg Steinhardt, Guenter Subklew, Tao Tao, Tingfeng Wu, Daqiang Yin, Fangfang Zhao, Binghui Zheng, Meiyue Zhou, Hua Zou, Jiane Zuo, Andreas Tiehm

**Affiliations:** 1 bbe moldaenke GmbH, Schwentinental, Germany; 2 CRAES: Chinese Research Academy of Environmental Sciences, Beijing, China; 3 DAHLEM Consultant Engineers, Darmstadt, Germany; 4 F.A.S.T. GmbH, Langenbrettach, Germany; 5 FZJ Forschungszentrum Jülich GmbH, Jülich, Germany; 6 GAIAC Research Institute for Ecosystem Analysis and Assessment at RWTH Aachen University, Aachen, Germany; 7 Hydroisotop GmbH, Schweitenkirchen, Germany; 8 IGB Leibniz-Institute of Freshwater Ecology and Inland Fisheries, Berlin, Germany; 9 inge GmbH, Greifenberg, Germany; 10 IWW Water Centre, Mulheim, Germany; 11 Jiangnan University, Wuxi, China; 12 KIT Karlsruhe Institute of Technology, Karlsruhe, Germany; 13 Leibniz University Hannover, Hannover, Germany; 14 NIGLAS: Nanjing Institute of Geography & Limnology, Chinese Academy of Sciences, Nanjing, China; 15 RWTH Aachen University, Aachen, Germany; 16 Steinhardt GmbH, Taunusstein, Germany; 17 Tongji University, Shanghai, China; 18 Tsinghua University, Beijing, China; 19 TZW Water Technology Center, Karlsruhe, Germany; 20 UFZ Helmholtz-Zentrum für Umweltforschung GmbH, Leipzig, Germany

**Keywords:** Water management, Urban catchment, Monitoring, Blue-green algae, Biodegradation, Isotope fractionation, Membrane filtration, Network flushing, Leakage detection, Governance

## Abstract

The Taihu (Tai lake) region is one of the most economically prospering areas of China. Due to its location within this district of high anthropogenic activities, Taihu represents a drastic example of water pollution with nutrients (nitrogen, phosphate), organic contaminants and heavy metals. High nutrient levels combined with very shallow water create large eutrophication problems, threatening the drinking water supply of the surrounding cities. Within the international research project SIGN (SinoGerman Water Supply Network, www.water-sign.de), funded by the German Federal Ministry of Education and Research (BMBF), a powerful consortium of fifteen German partners is working on the overall aim of assuring good water quality from the source to the tap by taking the whole water cycle into account: The diverse research topics range from future proof strategies for urban catchment, innovative monitoring and early warning approaches for lake and drinking water, control and use of biological degradation processes, efficient water treatment technologies, adapted water distribution up to promoting sector policy by good governance. The implementation in China is warranted, since the leading Chinese research institutes as well as the most important local stakeholders, e.g. water suppliers, are involved.

## Background

Water is essential for all forms of life. However, in many parts of the world, water quality is threatened by multiple pollution sources. In the last few decades, China has undergone rapid industrial and economic growth. Especially in densely populated areas, the need for clean water is constantly increasing [1]. At the same time, the quality of raw water is often impaired due to significant anthropogenic pollution [2, 3]. Furthermore, the available water resources per capita in China are naturally low (only one-quarter of the world average; [4]) and unevenly distributed, with high water scarcity in the dry northern parts of the country [5].

The Taihu (Tai lake) is located in the Yangtze Delta close to the megacity Shanghai in the provinces Jiangsu and Zhejiang. With an area of 2300 km^2^ [6], it is the third-largest freshwater lake in China [3]. The lake is only 2 m deep in average and it is connected to more than 220 rivers [7].

The Taihu region is one of the most economically prospering areas of China: with 0.38 % of the national land area and 3 % of the total national population, it produced 12 % of the GDP (gross domestic product) of China in 2010 [3]. Furthermore, the Taihu belongs to the focus regions of the currently running Chinese Major Program of Science and Technology for Water Pollution Control and Governance demonstrating its high scientific and political importance.

Due to its connection to a large number of inflowing rivers in a region with high anthropogenic activities, Taihu represents a drastic example of water pollution with nutrients (nitrogen, phosphate), organic contaminants and heavy metals [7, 8]. Improper handling of sewage and manure is one important cause of nitrate pollution [9]. Furthermore, a large number of organo chemicals from different sources can be detected such as pesticides from agriculture [10], pharmaceuticals from the surrounding cities [11] and perfluorinated compounds from industrial production and use [12]. Another water quality issue is the occurrence of unwanted microbes such as bacteria carrying antibiotic resistance genes [13, 14].

High nutrient levels combined with very shallow water create large eutrophication problems [6] and optimal growth conditions for cyanobacteria also known as blue-green algae. The resulting massive algal blooms are a large and often clearly visible problem of Taihu. Due to oxygen consumption and their toxin production, algal blooms are not only detrimental for the environment but also a large challenge for drinking water treatment. In 2007, two million inhabitants of Wuxi suffered for a week from discontinued tap water supply due to the heavily impaired raw water quality [15, 16].

The severity of environmental contamination in the Taihu region as well as in China in general is recognized since many years and led to a high number of research and management actions [5, 15, 17]. Despite numerous restoration efforts, the water quality of Taihu still remains impaired [6].

## Scope of the project—assure good water quality from the source to the tap

**Fig. 1:**
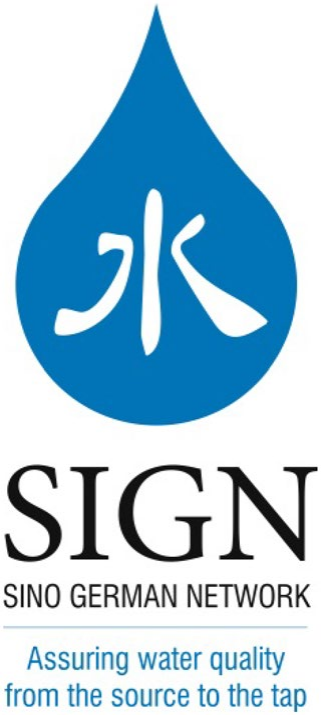
Logo of the SIGN project

The overall aim of the international research project SIGN (SinoGerman Water Supply Network, Fig. 1) is to assure good water quality from the source to the tap. Thus, the powerful consortium of fifteen German partners funded by the German Federal Ministry of Education and Research (BMBF) is working on the following topics taking the whole water cycle into account:

- Future proof strategies for urban catchment
- Monitoring and early warning
- Securing the quality of the lake as raw water resource
- Fostering the efficiency of water treatment
- Higher water quality and quantity through adapted water distribution
- Promoting sector policy by good governance
- Implementation of the results among the Chinese stakeholders

The close cooperation of the partners from academia and industry allows scientific progress while at the same time ensuring practical applicability of the newly developed technological solutions. German water technologies and management concepts are specifically developed and adapted to Chinese boundary conditions. The practical implementation in China is warranted, since the leading Chinese research institutes as well as the most important local stakeholders, e.g. water suppliers, are involved.

The SIGN project is working internationally as well as interdisciplinary. Thus, comprehensive exchange between the partners coming from different countries as well as from different disciplines is crucial in order to assure successful implementation of the research topics. The different work units, e.g. all partners involved with biodegradation, have frequent exchange via meetings and via email/telephone. Furthermore, all project partners as well as the concerned Chinese stakeholders encounter each other at large meetings at least three times during the project duration. The project is coordinated by the TZW—Water Technology Center—providing large experience with challenging multinational research projects as well as with fruitful SinoGerman cooperation e.g. within the Yangtze project [1, 18–20].

## Research topics—around the whole water cycle

### Future proof strategies for urban catchment

Responsible: DAHLEM Consultant Engineers, Leibniz University Hannover, RWTH Aachen University, Steinhardt GmbH.

Chinese partners: Beijing University of Civil Engineering and Architecture, Chinese Research Academy of Environmental Sciences (CRAES), Jiaxing Office of Comprehensive Water Control, Tsinghua University, Wuxi Drainage Co. Ltd.

#### Customized stormwater management with increased climate change resilience

The manifold challenges of sustainable urban drainage require a corresponding water sensitive inter-coordinated urban spatial and infrastructure planning. Different tasks such as provision of retention space, regulation of runoff in accordance with the sewerage system, easy maintenance and synergies between water, transport and energy infrastructures have to be fulfilled jointly. Thus, appropriate technical tools, guidance documents and administrative schemes are developed and tested. Overcoming the prevailing shortcomings in handling stormwater contributes to improved urban flood management. Furthermore, concrete climate adaption strategies are demonstrated.

#### Tailor-made sewer cleaning as one building block for future-proof wastewater management

The efficient operation and maintenance of urban drainage systems is another key aspect in reducing the number and frequency of wastewater and rainwater discharges into the receiving water bodies and improving the effluent quality of unavoidable sewer overflows. Inverted siphons and combined sewer overflows, known as important components and critical spots within drainage systems, are natural targets for upgrading [21]. Thus, for two such sites adapted cleaning devices are developed and tested under Chinese conditions. Checklists for operation and maintenance are provided and a risk assessment is carried out. In this way, German approaches can be transferred to Chinese boundary conditions in order to improve hydraulic structures and to assure integrity of sewer systems. An increased performance of the sewer system actively contributes to the prevention of urban floods.

### Monitoring and early warning

Responsible: Hydroisotop GmbH, KIT Karlsruhe Institute of Technology, GAIAC Research Institute for Ecosystem Analysis and Assessment at RWTH Aachen University, IWW Water Centre.

Chinese partners: Chinese Research Academy of Environmental Sciences (CRAES), Jiangnan University.

#### Innovative and automated monitoring methods for thorough assessment of lake water quality

Sensor and sampling technologies are advanced in order to study the spatial-temporal development of Taihu water quality [22, 23]. An innovative mobile water sensor and sampling system (Biofish) is adapted for the very specific shallow water conditions of Taihu. Furthermore, a new buoy system with a vertically moveable measuring platform is developed. The profiling buoy carries sampling devices as well as in situ and online multi-sensor equipment, e.g. algae sensors, for stationary and long-term water quality and meteorological measurements. The obtained data are evaluated in order to establish an early warning system for water quality.

#### Analysis of chemical pollution in view of suitable spots for drinking water abstraction

Water quality monitoring focuses on the water constituents that have special importance in view of drinking water production, as well as for the ecosystem. In addition to algae, toxicological, microbiological, inorganic (e.g. nitrogen, phosphorous, heavy metals) and organic parameters (e.g. industrial chemicals, pharmaceuticals, pesticides) are measured. Key pollutants are identified in order to facilitate water quality monitoring and to identify the specific sources of pollution. Recommendations for measures to mitigate the lake pollution as well as for optimized spots for drinking water abstraction are deduced.

#### Isotope measurements for the detection of pollution sources and turnover

Knowledge about the dynamic lake water balance and circulation in the Taihu is essential for the spatial-temporal evaluation of the lake water quality. Therefore, the H/O-isotope composition of lake and sediment water is evaluated together with climate and hydrological data. The C/N/(S)-isotope composition of water pollutants and their changes during biodegradation yield information about sources and transformations of nitrogen-species and dissolved organic carbon, algae growth as well as pollutant transport.

#### (Eco)toxicity testing to determine ecological and toxicological relevance of chemical pollutants

(Eco)toxicity on different trophic levels as well as mutagenic and endocrine effects are tested within lake water samples in order to correlate biological effects with the measured chemical pollution (e.g. [24, 25]). These tests are also applied to water samples taken during drinking water treatment in order to assess removal or possible formation of detrimental compounds during reactive treatment steps. In order to assess and predict an important source of toxicity of Taihu—the massive occurrence of blue-green algae—the stoichiometric lake model “StoLaM” is further developed to simulate algae growth.

### Securing the quality of the lake as raw water resource

Responsible: IGB Leibniz-Institute of Freshwater Ecology and Inland Fisheries, TZW Water Technology Center.

Chinese partners: Chinese Research Academy of Environmental Sciences (CRAES), Nanjing Institute of Geography & Limnology, Chinese Academy of Sciences (NIGLAS), Tongji University.

#### Biodegradation as useful ecosystem services and bacterial contaminants

Biodegradation of organic pollutants and nitrogen-containing compounds (nitrate, nitrite, ammonia) as beneficial ecosystem services is investigated by using modern PCR-techniques [19, 20] as well as by isotope fractionation [26–28] within laboratory degradation studies with original Taihu materials. Powerful PCR methods are also used to detect microorganisms having harmful effects such as bacteria with antibiotic resistance genes [29] as well as pathogenic viruses and bacteria, originating e.g. from faecal contaminations (microbial source tracking). The gained knowledge allows the development of mitigation strategies for chemical and microbial pollution.

#### Algae growth behaviour and prediction of algal blooms

Onset and duration of cyanobacteria blooms are controlled by the nutrient content [30] as well as by water disturbances induced e.g. by wind [31] and drainage of water from the Yangtze River. Vertical mixing leads to fluctuating light conditions encountered by the algae. Investigation and characterization of the decisive effects on algal growth as well as ecological modelling contribute to more precise forecasts of algal blooms, which are needed to optimize abstraction and treatment of drinking water.

### Fostering the efficiency of water treatment

Responsible: bbe Moldaenke GmbH, FZJ Forschungszentrum Jülich GmbH, inge GmbH.

Chinese partners: Suzhou Water Group Co. Ltd., Tongji University, Hua Yan Water Group.

#### Algae detectors analysing the total amount and the integrity of algal cells

Algae are measured within the lake itself in order to monitor their growth under different environmental conditions. Online analyses at the inlet of the drinking water treatment reveal changes in raw water quality. Further measurements at the different steps of drinking water treatment allow the assessment of the removal efficiencies for the algae themselves, their toxins as well as algae-released drinking-water-relevant taste & odour (T and O) compounds/precursors. New measuring tools are developed in order to assess the integrity status of algae cells and thus the risk of toxin release.

#### Elimination of algae during ultrafiltration of nutrient-rich raw water

Due to the impaired raw water quality, efficient solutions for drinking water treatment of algae contaminated water are needed. Ultrafiltration membranes are employed to efficiently remove algal cells and/or algal compounds. This reduces the formation of disinfection by-products and/or T and O compounds/precursors in subsequent oxidation steps within the drinking water treatment plant. The membrane itself is improved regarding material and geometry. Additionally, the operating modes are optimized with respect to energy-efficiency and maximum performance, supported by the data obtained from an algae-online analyser.

#### Oxidation techniques for the elimination of taste and odour compounds and their precursors

Precursors of T and O compounds are identified by in-depth analysis of raw water and water from different treatment steps. Formation of T and O compounds is investigated by mechanistic laboratory studies in order to optimize advanced oxidation processes. Improved elimination of drinking-water-relevant substances by technological process optimization using the above-mentioned ultrafiltration and advanced oxidation techniques is evaluated for practical application within drinking water treatment plants in China.

### Higher water quality and quantity through adapted water distribution

Responsible: F.A.S.T. GmbH, TZW Water Technology Center.

Chinese partners: Suzhou Water Group Co. Ltd., Tongji University, Hua Yan Water Group.

#### Adapted flushing strategies to remove deposits from the drinking water network

Drinking water leaving the waterworks in Suzhou has good and stable quality. However, water discoloration and problems with T and O compounds reoccur during transport through the drinking water pipes. In order to remove deposits and thus increase drinking water quality at the consumer’s tap, an innovative optimized flushing strategy is adapted and tested in a model region of the distribution network in Suzhou. Based on the deposit accumulation velocity, the needed flushing intervals are defined in order to allow deposit-free maintenance of the distribution network in the future.

#### Automated acoustic leak detection for drinking water pipes to minimize water losses

Tools for the automatic acoustic detection of leakages within the drinking water pipes are adapted and tested for a model region of the distribution network in Suzhou. The measuring data of the noise loggers are collected via wireless transmission, accessible via internet and evaluated with a specialized software tool. The gained knowledge about the occurring water losses helps the water suppliers to reduce the water losses from their water distribution networks to a minimum being obligatory for technical, hygienic as well as economic reasons.

### Promoting sector policy by good governance

Responsible: UFZ Helmholtz-Zentrum für Umweltforschung GmbH.

Chinese partners: Chinese Research Academy of Environmental Sciences (CRAES), Tongji University.

Achieving a sustainable urban environmental water cycle is not only a technical challenge. Feasibility, adequacy and performance of technical concepts are highly dependent on the socio-economic and institutional settings. Thus, recommendations for sustainable water policy and administration that consider the particular framework conditions in China are deduced. Further initiatives for effective participatory processes and awareness-raising campaigns are suggested.

### Implementation of the results among the Chinese stakeholders

Responsible: TZW Water Technology Center.

All results obtained are actively shared with the scientific community as well as with the concerned Chinese stakeholders e.g. via the website www.water-sign.de, with publications and with presentations at local and international conferences.

German best practice examples of pollution elimination and improvement of water resource quality are analysed in view of appropriateness for the Taihu region. Based on the existing Chinese standards and guidelines as well as on the raw water situation of Taihu, the most important parameters for drinking water quality are selected in order to facilitate future monitoring and control.

## Abbreviations

BMBF: German Federal Ministry of Education and Research
CLIENT: International Cooperations for Sustainable Innovations
GDP: gross domestic product
SIGN: SinoGerman Water Supply Network
T&O: taste and odour
